# Supplementary material for: Profiling of Steroid Metabolic Pathways in Human Plasma by GC-MS/MS Combined with Microwave-Assisted Derivatization for Diagnosis of Gastric Disorders
Source: Int J Mol Sci. 2021 Feb 13;22(4):1872. doi: 10.3390/ijms22041872 (PMC7918215; doi:10.3390/ijms22041872)
Supplement: Supplementary file 1 [file ijms-22-01872-s001.pdf]

Supplementary Materials

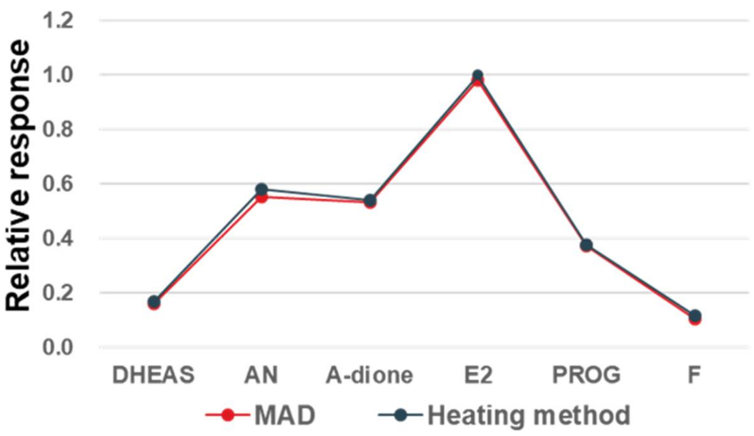

Figure S1. Comparison of relative response for 6 steroid derivatives using MAD and traditional thermal block heating.

Table S1. List of dMRM transitions and parameters for steroid analysis.

Compound Name	Retention Time (min)	Quantifier ion Transition	CE	Qualifier ion Transition	CE
AN	6.22	434 > 419	6	419 > 239	12
DHEA	6.40	432 > 417	6	417 > 327	6
Adiol	6.69	329 > 239	9	344 > 239	15
7α-OH-DHEA	6.70	325 > 235	9	415 > 325	9
DHEAS	4.65	327 > 237	6	342 > 327	9
DHT	6.80	434 > 405	3	434 > 377	6
Adione	6.95	430 > 209	20	430 > 415	9
T	7.15	432 > 209	20	432 > 417	12
T-d_5	7.10	436 > 73	27	436 > 213	18
E1	6.86	414 > 399	9	414 > 155	15
E2	7.10	416 > 285	12	285 > 205	15
E3	9.88	345 > 73	27	504 > 414	3
E2-d_5	7.03	287 > 287	3	287 > 73	15
Preg	9.16	445 > 157	18	445 > 265	6
Prog	9.80	458 > 157	15	458 > 443	9
17α-OH-Preg	11.72	548 > 548	3	230 > 215	6
17α-OH-Prog	10.86	546 > 546	3	316 > 208	15
Prog-d_9	9.78	465 > 160	24	465 > 450	18
Chol	13.50	329 > 329	3	368 > 353	9
7α-OH-Chol	14.02	456 > 456	3	456 > 233	15
Chol-d_7	13.29	359 > 359	3	333 > 333	3
21-DF	14.53	634 > 634	3	404 > 208	12
E	19.12	615 > 615	3	630 > 630	3
F	19.15	632 > 632	3	632 > 191	27
F-d_4	18.46	636 > 636	3	636 > 546	9
Phenanthrene-d_{10}	2.30	188 > 188	3	188 > 160	20

Table S2. Summary of information for enrolled patients with different gastric disorders.

**Samples**		**CSG**	**IM**	**Gastric Cancer**
Sex	Male	8	8	23
	Female	12	5	10
Age		53.22 ± 17.03	56.93 ± 11.92	62.59 ± 11.09

Table S3. Summary of method validation results for 20 steroid hormones.

Analytes	Calibration Range	Linear regression Equation	r^2	LODs (ng/mL)	LOQs (ng/mL)	Spiked Concentration (ng/mL)	Intra-Day Assay (%)	Inter-Day Assay (%)
AN	6-2000	y=0.002x-0.0232	0.9996	0.001	0.004	6	99.98 (3.81)	100.01(2.98)
						250	99.01 (3.84)	99.65(3.53)
						1000	102.52 (6.49)	102.19(2.31)
DHEA	6-2000	Y=0.0019x-0.0233	0.9990	0.012	0.039	6	99.99 (1.13)	99.99(3.46)
						250	98.65 (4.81)	99.77(5.33)
						1000	102.57 (6.15)	102.12(1.52)
A-diol	6-2000	y=0.0015x-0.0263	0.9989	0.004	0.013	6	99.99 (7.08)	100.01(7.5)
						250	99.09 (6.69)	99.80(3.18)
						1000	101.32 (7.91)	100.75(1.82)
7α-OH-DHEA	6-2000	y=0.002x-0.0354	0.9991	0.002	0.007	6	99.99 (5.46)	99.99(4.96)
						250	98.97 (5.93)	99.82(4.03)
						1000	99.99 (7.03)	101.45(7.93)
DHEAS	6-2000	y=0.0002x+0.0007	0.9941	0.175	0.583	20	104.99 (5.85)	94.89 (9.84)
						250	114.73 (7.44)	105.02 (12.45)
						1000	115.47 (13.19)	117.04(3.43)
A-dione	6-2000	y=0.0017x-0.0172	0.9995	0.002	0.006	6	99.99 (3.97)	100.02(4.77)
						250	98.68 (4.85)	102.41(7.16)
						1000	102.52 (3.04)	100.01(2.10)
DHT	6-2000	y=0.0007x-0.0064	0.9992	0.012	0.040	6	99.99 (3.58)	100.01(6.98)
						250	99.68 (1.71)	100.31(3.37)
						2000	101.18 (5.66)	100.78(1.37)
T	6-2000	y=0.0022x-0.0659	0.9925	0.001	0.004	6	99.99 (0.48)	100.01 (3.95)
						250	99.05 (3.62)	99.63 (3.37)
						2000	99.25 (6.75)	99.49 (1.17)
E1	6-2000	y=0.0045x-0.1822	0.9941	0.001	0.003	6	100.01 (5.66)	99.99(5.11)
						250	99.34 (3.20)	99.90(1.19)
						1000	101.66 (2.77)	100.94(1.22)

E2	6-2000	y=0.0038x-0.1223	0.9958	0.001	0.003	6	99.99 (6.17)	99.99(2.82)
						250	98.98 (4.92)	99.78(1.42)
						1000	101.66 (1.05)	101.01(1.12)
E3	6-2000	y=0.0045x-0.1822	0.9941	0.225	0.751	6	99.99 (4.39)	99.99(9.48)
						250	99.07 (7.90)	99.85(5.10)
						1000	101.39 (3.13)	101.21(1.12)
PREG	6-2000	y=0.0023x-0.0435	0.9992	0.001	0.003	6	99.99 (3.41)	100.01(8.75)
						250	98.90 (3.50)	99.77(2.97)
						1000	102.26 (11.30)	102.06(6.86)
PROG	6-2000	y=0.0023x-0.0633	0.9965	0.001	0.004	6	100.01 (1.90)	100.01(3.90)
						250	99.23 (3.01)	99.85(4.28)
						1000	100.33 (8.71)	100.09(5.47)
17α-OH-PREG	6-2000	y=0.0024x+0.0301	0.9953	0.048	0.159	6	99.99 (11.38)	100.09(17.25)
						250	97.88 (4.35)	99.17(7.87)
						2000	99.11 (3.51)	98.98(3.15)
17α-OH-PROG	6-2000	y=0.0019x-0.0903	0.9932	0.031	0.104	6	100.01 (4.90)	99.99(16.50)
						250	99.80 (1.85)	100.01(8.14)
						2000	98.53 (2.24)	101.80(5.69)
Chol	6-2000	y=0.0009x+0.0177	0.9980	0.071	0.197	6	99.96 (12.16)	99.99(15.29)
						250	100.54 (5.22)	100.48(9.12)
						1000	98.68 (7.80)	99.48(19.93)
7α-OH-Chol	6-2000	y=0.0049x-0.0795	0.9994	0.001	0.002	6	99.96 (9.54)	99.82(4.05)
						250	98.99 (6.03)	99.42(14.06)
						2000	106.73 (8.42)	99.91(6.02)
21-DF	6-2000	y=0.0031x-0.1669	0.9970	0.007	0.024	6	99.99 (7.58)	99.99(7.10)
						250	100.54 (2.59)	100.34(12.85)
						2000	104.99 (7.49)	102.76(5.66)
E	6-2000	y=0.0034x-0.1056	0.9984	0.075	0.023	6	100.01 (5.15)	99.99(9.21)
						250	101.04 (3.06)	100.59(12.04)
						2000	99.21 (7.34)	104.90(3.49)
F	6-2000	y=0.0063x-0.589	0.9949	0.337	1.125	20	100.01 (5.14)	99.99(4.99)
						500	99.66 (5.49)	100.01(4.45)
						1000	97.38 (6.72)	99.43(5.57)
